# Risk factors for hypocalcemia in dialysis patients with refractory secondary hyperparathyroidism after parathyroidectomy: a meta-analysis

**DOI:** 10.1080/0886022X.2022.2048856

**Published:** 2022-03-13

**Authors:** Dan Gao, Yan Lou, Yingchun Cui, Shengmao Liu, Wenpeng Cui, Guangdong Sun

**Affiliations:** Department of Nephrology, The Second Hospital of Jilin University, Changchun, China

**Keywords:** Secondary hyperparathyroidism, risk factors, hypocalcemia, meta-analysis

## Abstract

**Objective:**

Hypocalcemia after parathyroidectomy (PTX) results in tetany, diarrhea, cardiac arrhythmia, and even sudden death. However, a meta-analysis or systematic evaluation of risk factors with the occurrence and development of hypocalcemia in patients with secondary hyperparathyroidism (SHPT) after PTX has never been performed.

**Methods:**

A thorough search of electronic databases, including PubMed, Web of Science, the Cochrane Library, and EMBASE, was performed to retrieve relevant studies from database inception to June 2021. Quality of the included studies was assessed by two independent reviewers using the Newcastle–Ottawa Scale. Review Manager 5.3 and Stata 16.0 were used for meta-analysis. The random-effects model was adopted to calculate the 95% CIs (*I*^2^> 50% or *p* < 0.05) of the combined effect size and the corresponding homogeneous data. Otherwise, a fixed-effects model was used.

**Results:**

Thirteen studies including 2990 participants who met the inclusion criteria were enrolled in the present meta-analysis. The overall quality of the enrolled studies had a score of >7 points. Risk factors significantly related to hypocalcemia in patients with SHPT after PTX were preoperative serum calcium (OR 0.19, 95%CI 0.11–0.31), preoperative alkaline phosphatase (ALP) (OR 1.01, 95% CI 1.01–1.02), and preoperative intact parathyroid hormone (iPTH) (OR 1.38, 95%CI 1.20–1.58). Meanwhile, age (OR 0.97, 95%CI 0.87–1.10) was not significantly correlated with hypocalcemia after PTX.

**Conclusions:**

Based on the current evidence, preoperative serum calcium, preoperative ALP, and preoperative iPTH were significant predictors of hypocalcemia in patients with SHPT after PTX. More attention should be given to patients with these risk factors for the prevention of postoperative hypocalcemia.

## 1. Introduction

Secondary hyperparathyroidism (SHPT) is a common complication in patients with end-stage kidney disease (ESKD). Hyperplasia of the parathyroid glands and abnormal increase in intact parathyroid hormone (iPTH) secretion are the primary manifestations of SHPT [1]. Complications often seen in this condition include refractory pruritus, bone pain, muscular weakness, progressive soft tissue calcification, kidney stones, constipation, peptic ulcer disease, spontaneous long bone fracture, psychosis, dementia, and even risk of death [2–4]. Control of SHPT in the early stages is achieved through medical therapies including calcium supplements, vitamin D supplements, phosphate-poor diets, phosphate-binders, and the calcimimetic agents [5–7]. Despite the emergence of new therapeutic agents, most guidelines still recommend PTX as the treatment of choice in SHPT patients’ refractory to medical therapy. PTX can effectively decrease iPTH, relieve bone pain, improve quality of life, and reduce complications and mortality rates.

Hypocalcemia or even hungry bone syndrome (HBS) after PTX is a well-known and severe complication [5,8–10] reported to affect most patients with chronic kidney disease (CKD). Hypocalcemia may trigger weakness, headache, paresthesia, muscle cramps, laryngeal stridor, seizures, cardiac arrhythmias, and tetany. In severe situations, hypocalcemia may even cause sudden death [11]. Thus, it is beneficial and clinically significant to identify the high-risk patient group susceptible to postoperative hypocalcemia for more vigilant postoperative monitoring [5].

At present, several reports have explored the risk factors for hypocalcemia in patients with SHPT after PTX. However, results from these studies are controversial due to the small sample size and limited outcome indicators. This meta-analysis enrolled 13 eligible studies to analyze and compare the risk factors for hypocalcemia and non-hypocalcemia after PTX, with the aim of providing more reliable evidence for clinical practice.

## 2. Methods

### 2.1. Data collection

A thorough search of electronic databases, including the Cochrane Library, EMBASE, PubMed, and Web of Science was performed. Relevant articles published till June 2021 related to the risk factors for hypocalcemia in patients with SHPT after PTX were identified. The search terms used were [Hyperparathyroidism, Secondary OR Secondary Hyperparathyroidism OR Hyperparathyroidisms, Secondary OR Secondary hyperparathyroidisms] AND [Risk Factors OR Factor, Risk OR Risk Factor OR Health Correlates OR Correlates, Health OR Risk Scores OR Risk Score OR Score, Risk OR Risk Factor Scores OR Risk Factor Score OR Score, Risk Factor OR Population at Risk OR Populations at Risk] (Supplementary Documents). All the confirmed works were imported into EndNote X20.1 for management. An additional search for the reference lists in the included studies and recent reviews or meta-analyses was done.

### 2.2. Inclusion criteria

The inclusion criteria for the study are as follows: (a) the study was a case-control or cohort study; (b) the study focused on the risk factors for hypocalcemia in patients with SHPT after PTX; (c) patients at the CKD-5D stage who met the diagnostic criteria for refractory SHPT and underwent PTX were also considered; (d) the surgical methods included total PTX, subtotal PTX, with or without parathyroid autotransplantation, or ultrasound-guided microwave ablation (MWA); and (e) the outcomes reported were adjusted or non-adjusted odds ratios (ORs) and 95% confidence intervals (95% CI) on multiple logistic regression analysis. Hypocalcemia after surgery was defined as the minimum serum calcium level lower than 2.1 mmol/L (8.4 mg/dL) within three days postoperatively. The search was limited to English language manuscripts.

### 2.3. Exclusion criteria

Studies that met the following criteria were excluded: (a) duplicate studies; (b) reports, reviews, or meeting abstracts; (c) no diagnostic criteria for hypocalcemia; and (d) insufficient data for the calculation of ORs and 95% CIs.

### 2.4. Data extraction

The baseline information were extracted and evaluated by two reviewers (Dan Gao and Yan Lou). Any disagreement between the reviewers was resolved through mutual discussion. Data extracted included author, year of publication, study time and country, study design, number of cases and controls, and data on risk factors. Moreover, the extracted risk factors were age, male sex, body weight, preoperative serum calcium, preoperative ALP, preoperative iPTH, preoperative serum phosphorus, pruritus, preoperative serum albumin, weight of resected glands, and volume of resected glands.

### 2.5. Quality assessment

Quality assessment of the enrolled studies was done independently by two reviewers (Dan Gao and Yan Lou) using the Newcastle–Ottawa Scale (NOS) [12]. The NOS consists of three parts namely four points for selection, two points for comparability, and three points for outcome and exposure. Studies were then classified according to their quality into poor (0–4), moderate (5–6), or high quality (7–9). Only high-quality studies were included in the meta-analysis.

### 2.6. Statistical analysis

Review Manager 5.3 (Cochrane Cooperative in Copenhagen, Denmark) was used for statistical analysis of data. The risk factors for hypocalcemia in patients with SPTH after PTX were assessed through calculation of pooled ORs. The Z-test was used to determine the significance of differences. Heterogeneity in the analysis was evaluated using the χ^2^ test and *I*^2^ statistic. In addition, the random-effects model was adopted to calculate the 95% CIs (*I*^2^> 50% or *p* < 0.05) of the combined effect size and the corresponding homogeneous data. Otherwise, a fixed-effects model was used. Sensitivity analysis was performed by omitting one study each time and exchanging the effect models. The result was considered robust if no significant difference was observed in the *P*-value of the corresponding combined effect size. Moreover, Egger’s test of Stata 16.0 (StataCorp, College Station, TX) was used to check for publication bias. A *p*-value  > 0.05 was obtained, indicating that there was no publication bias in this study. Typically, the combined exposure rate of the control was used to replace the exposure rate of the overall population.

## 3. Results

### 3.1. Study selection

**Figure 1.**
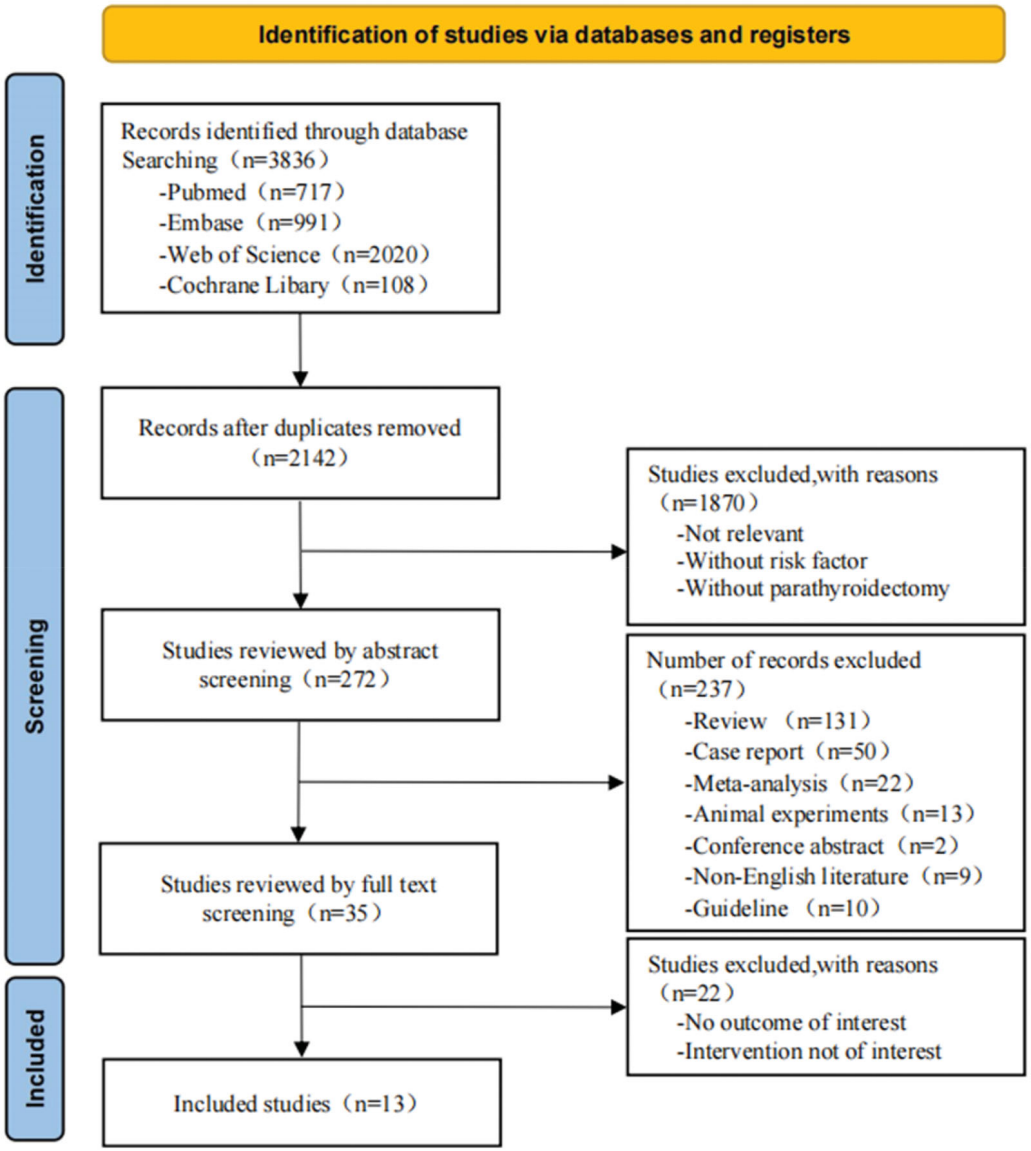
The flow diagram of the study identification and inclusion and exclusion process.

A total of 3836 potentially relevant studies were discovered by searching the electronic databases, 2142 of which were excluded due to duplication, 1870 were excluded after title screening, 237 were excluded after abstract screening, and 22 were excluded due to inability to meet the inclusion criteria after a full-text review. Finally, 13 studies were included in the meta-analysis. The flowchart of the study selection process is presented in Figure 1.

### 3.2. Study characteristics

In total, 13 studies published from 2013 to 2021 involving 2990 participants (1739 patients and 1251 controls) that investigated 11 risk factors were included in the study. Of these, nine were published in China, while the other four originated from Thailand, Tunisia, Mexico, and Malaysia. Eleven of these studies were case-control studies, whereas two were cohort studies. There were complete basic research data on factors including preoperative serum calcium, preoperative ALP, preoperative iPTH, and age, which were mentioned in more than three studies. However, other risk factors such as preoperative serum phosphorus, pruritus, preoperative serum albumin, weight of resected glands, body weight, male sex, and volume of resected glands were not analyzed in detail. This is due to the lack of original data or limited references which could possibly lead to bias. Two of the cohort studies were grouped, which might be considered as two groups of analysis data. Specific information of studies included in this meta-analysis is presented in Table 1.

**Table 1.** The general characteristics of the included studies.

Study	Year	Country	Design	Study period	Sample size	Case/controls	Surgical method	Hypocalcemiastandard (mmol/L)	Risk factors
Guang Yang	2018	China	Case-control	2013.01–2017.04	252	180/72	PTX + AT	<2.000	①③
Jorge l.Fonseca-Correa	2021	Mexico	Case-control	2003.10–2019.10	87	67/20	PTX	<1.870	③⑩
KittraweeKritmetapak	2020	Thailand	Case-control	2014.01–2020.01	130	107/23	PTX	<2.100	①③④⑥
Lo-Yi Ho	2017	China	Case-control	2004.01–2014.02	62	17/45	T-PTX	<2.100	①⑥⑨
M.Hamouda	2013	Tunisia	Case-control	–	70	48/22	PTX	<2.000	③⑥⑦
Mingjun Wang	2020	China	Case-control	2016.10–2018.10	114	87/27	PTX + AT	<2.100	①③④⑧
Ping Wen	2020	China	Case-control	2008.01–2018.12	1095	811/284	T-PTX	<1.780	③④
Poh Guan Tan	2020	Malaysia	Case-control	2007.01–2014.12	68	25/43	PTX	<2.000	③⑥
Wan-Chuan Tsai	2015	China	Case-control	2001.02–2012.09	420	157/263	PTX	<1.875	②③④
Wei Gong	2021	China	Case-control	2013.05–2020.02	87	18/69	PTX + AT	<2.100	③④⑥
Yifei Ge	2019	China	Case-control	2015.07–2017.12	115	101/14	PTX + AT	<2.100	①③
Ying Wei (reference #8)	2020	China	Cohort	2018.03–2019.05	286	76/210	WMA/TPTX	<1.875	WMA:①③⑦
									TPXT:①③⑪
Ying Wei (reference #16)	2020	China	Cohort	2015.07–2018.05	204	45/159	MWA	<1.875	model.1:①④⑧
									model.2:①④⑧

PTX: parathyroidectomy; T-PTX:total-parathyroidectomy;PTX + AT: parathyroidectomy with forearm autograft; MWA: microwave ablation. ①preoperative serum calcium ②preoperative serum phosphorus ③preoperative ALP ④preoperative intact parathormone ⑤Pruritus ⑥Age ⑦preoperative serum albumin ⑧Weight of resected glands ⑨Body weight ⑩Males ⑪Volume of resected glands.

### 3.3. Assessment of study quality

All studies were considered high-quality, as evaluated by NOS (Table 2).

**Table 2.** Quality assessment of studies included in this meta-analysis by Newcastle-Ottawa Scale.

Study	Year	Selection	Comparability	Exposure/Outcome	Quality evaluation
Guang Yang	2018	★★★★	★★	★★	8
Jorge l.Fonseca-Correa	2021	★★★	★	★★★	7
KittraweeKritmetapak	2020	★★★★	★	★★★	8
Lo-Yi Ho	2017	★★★	★★	★★	7
M.Hamouda	2013	★★★	★★	★★	7
Ying Wei (reference #16)	2020	★★★★	★★	★★	8
Mingjun Wang	2020	★★★★	★	★★	7
Ping Wen	2020	★★★★	★	★★★	8
Poh Guan Tan	2020	★★★	★★	★★★	8
Wan-Chuan Tsai	2015	★★★	★★	★★	7
Wei Gong	2021	★★★	★★	★★★	8
Yifei Ge	2019	★★★	★★	★★★	8
Ying Wei (reference #08)	2020	★★★	★★	★★	7

### 3.4. Results of meta-analysis

#### 3.4.1. Preoperative serum calcium

**Figure 2.**
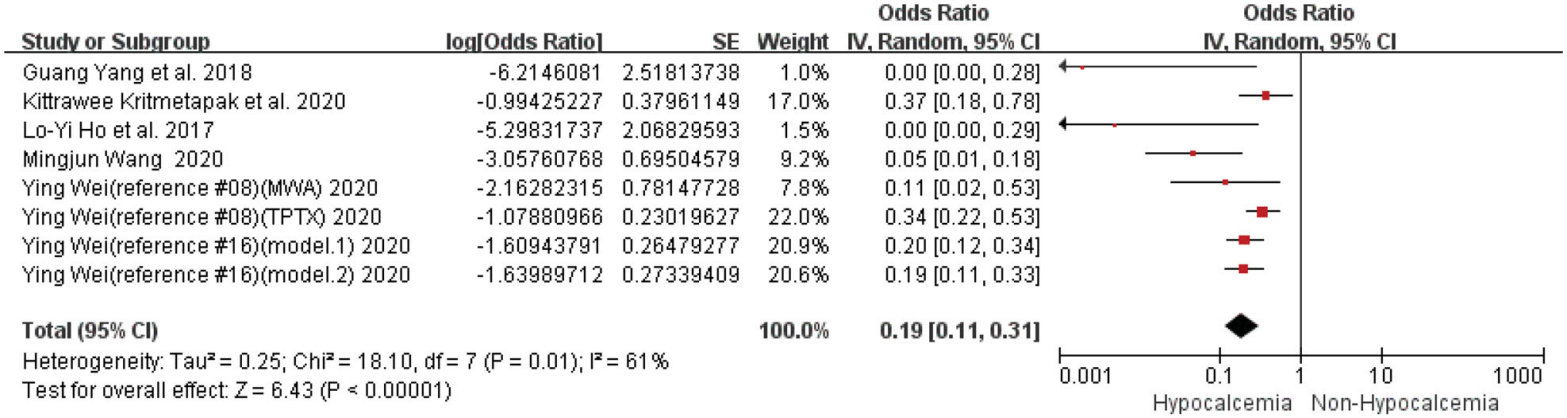
Forest plot of preoperative serum calcium.OR = odds ratio.

Results of preoperative serum calcium were reported in six studies (including eight groups of data) [8,13–17]. The statistical heterogeneity was high (I^2^ = 61%, *p* = 0.01), and a random-effects model was used to conduct a meta-analysis on the unadjusted data. The results showed a negative correlation between preoperative serum calcium levels and hypocalcemia after PTX. Simply put, preoperative hypocalcemia was a risk factor for postoperative hypocalcemia, and the results of the meta-analysis were statistically significant [OR = 0.19, 95%CI (0.11,0.31)] (*p* < 0.00001) (Figure 2). In addition, individual studies were separately excluded for the sensitivity analysis. The results revealed no significant difference in the meta-analysis results before and after excluding one single study, indicating the stability of the pooled results (Table 3).

**Table 3.** Sensitivity analysis for the meta-analysis of preoperative serum calcium by excluding one study at a time.

Study	Results of meta-analysis		Heterogeneity of study design		
	OR (95%CI)	*p* Value	χ^2^	*p* Value	*I*^2^(%)
Original Meta Study	0.19 (0.11,0.31)	<0.00001	18.10	0.01	61
(delete)Guang Yang	0.20 (0.13,0.32)	<0.00001	14.53	0.02	59
(delete)Lo-Yi Ho	0.20 (0.13,0.33)	<0.00001	14.65	0.02	59
(delete)Ying Wei (#16) (model.1)	0.17 (0.09,0.33)	<0.00001	17.70	0.007	66
(delete)Ying Wei (#08)(MWA)	0.19 (0.11,0.33)	<0.00001	17.28	0.008	65
(delete)Ying Wei (#08)(TPTX)	0.16 (0.09,0.28)	<0.00001	13.94	0.03	57
(delete)KittraweeKritmetapak	0.16 (0.09,0.29)	<0.00001	16.36	0.01	63
(delete)Ying Wei (#16) (model.2)	0.17 (0.09,0.33)	<0.00001	17.56	0.007	66
(delete)Mingjun Wang	0.22 (0.14,0.36)	<0.00001	12.64	0.05	53

#### 3.4.2. Age

**Figure 3.**
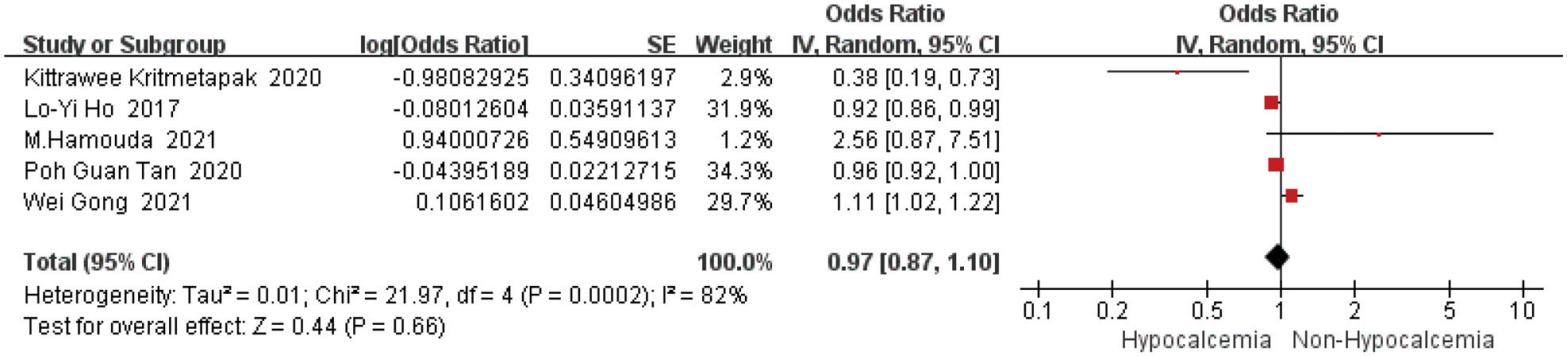
Forest plot of age. OR = odds ratio.

In total, five studies mentioned the results of age [5,14,15,18,19]. The statistical heterogeneity was high (I^2^=82%, *p* = 0.0002), and a random-effects model was used to perform a meta-analysis was performed on the unadjusted data. Results from the analysis show no statistical difference in the pooled results [OR = 0.97, 95%CI (0.87,1.10)] (*p* = 0.66) (Figure 3). Individual studies were later eliminated one at a time for sensitivity analysis (Table 4). Although most of the data did not change, the statistical heterogeneity was reduced to 74% upon removal of the study by Gong (*p* = 0.01). Therefore, a random-effects model was used for meta-analysis, and the pooled results revealed no statistical difference [OR = 0.93, 95%CI (0.82,1.05)] (*p* = 0.22). This suggests that age is not a risk factor for hypocalcemia after PTX.

**Table 4.** Sensitivity analysis for the meta-analysis of age by excluding one study at a time.

Study	Results of meta-analysis		Heterogeneity of study design		
	OR (95%CI)	*p* Value	χ^2^	*p* Value	*I*^2^(%)
Original Meta Study	0.97 (0.87,1.10)	0.66	21.97	0.00002	82
(delete)KittraweeKritmetapak	1.00 (0.90,1.10)	0.95	14.21	0.003	79
(delete)Lo-Yi Ho	0.98 (0.81,1.20)	0.86	19.66	0.0002	85
(delete)M.Hamouda	0.97 (0.86,1.08)	0.54	18.83	0.0003	84
(delete)Poh Guan Tan	0.96 (0.76,1.21)	0.71	21.26	<0.0001	86
(delete)Wei Gong	0.93 (0.82,1.05)	0.22	11.39	0.010	74

#### 3.4.3. Preoperative ALP

**Figure 4.**
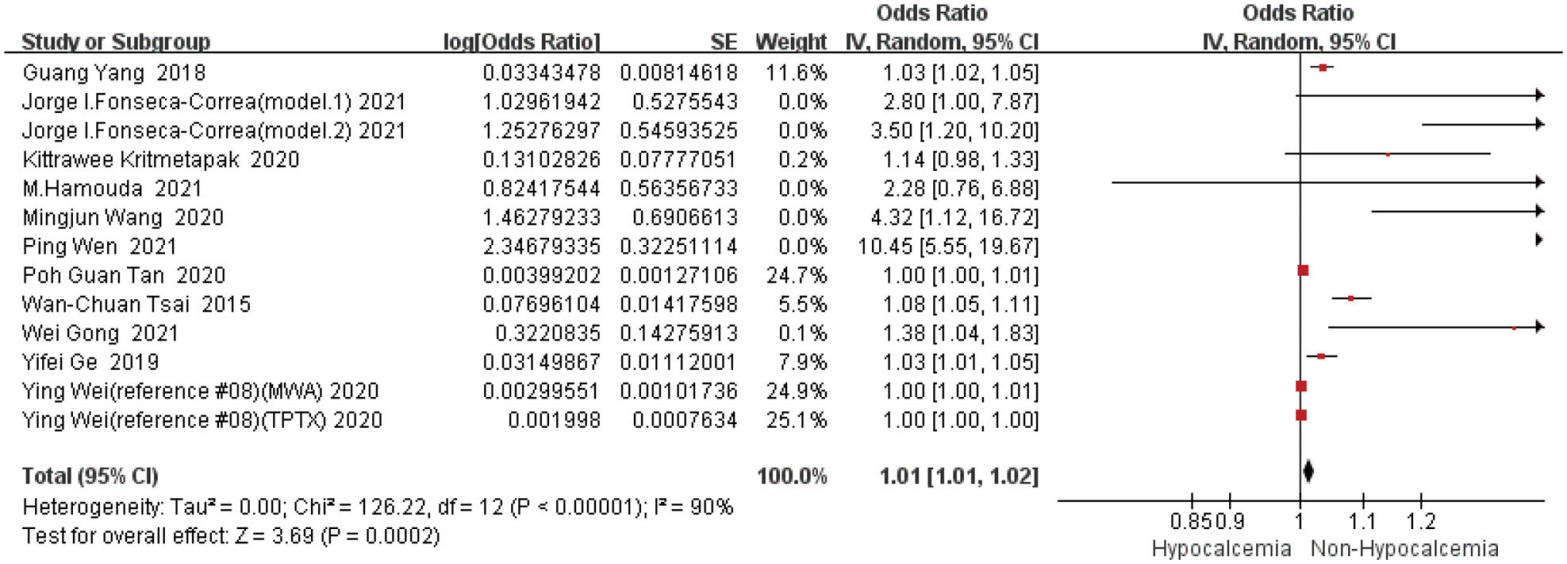
Forest plot of preoperative ALP.OR = odds ratio.

A total of 11 studies (including 13 groups of data) provided the results of preoperative ALP [2,5,8,13,14,17–22]. The statistical heterogeneity was high with I^2^=90% (*p* < 0.00001). Therefore, a meta-analysis was conducted on the unadjusted data using a random-effects model. According to the results, preoperative ALP was a risk factor for postoperative hypocalcemia, and results of the meta-analysis were statistically significant [OR = 1.01, 95%CI (1.01,1.02)] (*p* = 0.0002) (Figure 4). Individual studies were later excluded one at a time for sensitivity analysis. The results revealed no significant difference in the meta-analysis results before and after elimination of one single study, indicating the stability of the pooled results (Table 5).

**Table 5.** Sensitivity analysis for the meta-analysis of preoperative ALP by excluding one study at a time.

Study	Results of meta-analysis		Heterogeneity of study design		
	OR (95%CI)	*p* Value	χ^2^	*p* Value	I^2^(%)
Original Meta Study	1.01 (1.01,1.02)	0.0002	126.22	<0.00001	90
(delete)Guang Yang	1.01 (1.00,1.02)	0.005	112.20	<0.00001	90
(delete)Jorge l.Fonseca-Correa(model.1)	1.01 (1.01,1.02)	0.0002	122.43	<0.00001	91
(delete)Jorge l.Fonseca-Correa(model.2)	1.01 (1.01,1.02)	0.0002	120.98	<0.00001	91
(delete)KittraweeKritmetapak	1.01 (1.01,1.02)	0.0003	123.51	<0.00001	91
(delete)M.Hamouda	1.01 (1.01,1.02)	0.0002	124.09	<0.00001	91
(delete)Mingjun Wang	1.01 (1.01,1.02)	0.0002	121.75	<0.00001	91
(delete)Ping Wen	1.01 (1.00,1.02)	0.0004	73.4	<0.00001	85
(delete)Poh Guan Tan	1.02 (1.01,1.03)	<0.0001	125.48	<0.00001	91
(delete)Wan-Chuan Tsai	1.01 (1.00,1.02)	0.008	98.96	<0.00001	89
(delete)Wei Gong	1.01 (1.01,1.02)	0.0003	121.22	<0.00001	91
(delete)Yifei Ge	1.00 (1.00,1.00)	0.002	119.64	<0.00001	91
(delete)Ying Wei(#08)(MWA)	1.02 (1.01,1.03)	<0.0001	126.22	<0.00001	91
(delete)Ying Wei(#08)(TPTX)	1.02 (1.01,1.04)	<0.0001	122.62	<0.00001	91

#### 3.4.4. Preoperative iPTH

**Figure 5.**
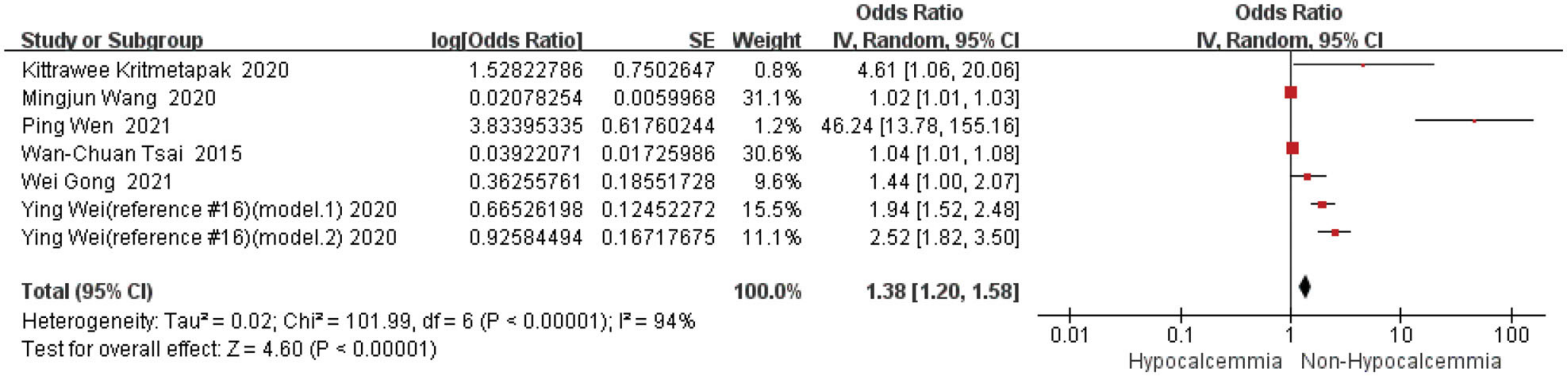
Forest plot of preoperative iPTH. OR = odds ratio.

In total, six studies (including seven groups of data) reported the results of preoperative iPTH^2^ [14,16,17,19,21],. Due to the high statistical heterogeneity (I^2^=94%) (*p* < 0.00001), a random-effects model was utilized for the meta-analysis of the unadjusted data. As revealed by the results, preoperative iPTH level was a risk factor for postoperative hypocalcemia, and the meta-analysis results were statistically significant [OR = 1.38, 95%CI (1.20,1.58)] (*p* < 0.00001) (Figure 5). Individual studies were then eliminated one at a time for the sensitivity analysis. Results show that there was no significant difference in the meta-analysis results before and after eliminating one study, indicating the stability of the pooled results (Table 6).

**Table 6.** Sensitivity analysis for the meta-analysis of preoperative iPTH by excluding one study at a time.

Study	Results of meta-analysis		Heterogeneity of study design		
	OR (95%CI)	*p* Value	χ^2^	*p* Value	*I*^2^(%)
Original Meta Study	1.38 (1.20,1.58)	<0.00001	101.99	<0.00001	94
(delete)KittraweeKritmetapak	1.36 (1.19,1.55)	<0.00001	97.98	<0.00001	95
(delete)Ying Wei (#16) (model.1)	1.25 (1.09,1.42)	0.001	75.56	<0.00001	93
(delete)Ying Wei (#16) (model.2)	1.23 (1.09,1.40)	0.001	72.97	<0.00001	93
(delete)Mingjun Wang	2.51 (1.47,4.29)	0.0008	95.60	<0.00001	95
(delete)Ping Wen	1.26 (1.12,1.41)	<0.0001	63.96	<0.00001	92
(delete)Wan-Chuan Tsai	2.51 (1.46,4.33)	0.0009	101.31	<0.00001	95
(delete)Wei Gong	1.37 (1.18,1.57)	<0.0001	98.69	<0.00001	95

### 3.5. Publication bias of the included studies

Egger’s test was used to evaluate the publication bias of the studies enrolled in the meta-analysis. The results revealed no obvious evidence of publication bias (*Z* = 0.31, *p* = 0.755 > 0.05) (Supplementary Figure 1).

## 4. Discussion

This study explored the risk factors of postoperative hypocalcemia after PTX. Although postoperative hypocalcemia is a well-known severe complication after PTX [23–25], the incidence rate of hypocalcemia varies greatly between studies, with postoperative hypocalcemia among patients with SHPT reported to range from 26% to 95% [18,23,26,27]. The main reasons for this observation are as follows: first, the definition of hypocalcemia varied among the enrolled studies. Second, the definition of HBS in some studies was based solely on serum calcium concentration immediately after PTX. However, it is important to note that some patients with HBS experience substantially reduced postoperative serum calcium concentrations from baseline. Moreover, clinical symptoms may develop despite these concentrations falling within the normal range. Therefore, the latter definition of HBS may have limited validity and lead researchers to underestimate its actual incidence [14]. In other words, hypocalcemia may develop at a later time, regardless of the modest drop in serum calcium level early after surgery [15]. Third, the parathyroid procedures performed are rather variable in these studies, including subtotal PTX, total PTX with autotransplantation, total PTX, and radiofrequency ablation [15]. The selected operative method definitely affects the postoperative serum calcium content. In example, patients who underwent subtotal PTX will have a slightly higher postoperative serum calcium level than those receiving total PTX. Fourth, preoperative interventions such as the type of dialysis, time on dialysis, calcimimetics, vitamin D supplementations, and calcium tablets may also affect outcomes.

Multiple factors are involved in the pathogenesis of postoperative hypocalcemia. A total of 11 risk factors associated with postoperative hypocalcemia were identified in these 13 studies, including preoperative serum calcium, phosphorus, ALP, iPTH, ALB, pruritus, age, weight of resected glands, body weight, male sex, and volume of resected glands. Among those mentioned, four risk factors were involved in more than three studies namely preoperative serum calcium, age, ALP, and iPTH. Therefore, a meta-analysis of these four risk factors was performed in this study. None of the remaining factors evaluated were predictive of the development of postoperative hypocalcemia in patients with SHPT after total PTX.

Hypoparathyroidism after PTX results in a rapid shift of calcium from the circulation to the skeletal system. Therefore, patients with preoperative hypocalcemia are more likely to develop hypocalcemia after PTX [28,29]. A higher preoperative serum calcium level predicts more active bone remodeling, a greater risk of postoperative HBS, and a higher risk of hypocalcemia after PTX. This meta-analysis concluded that preoperative hypocalcemia was a risk factor for hypocalcemia after PTX. The results of this study strongly suggest that appropriate calcium supplement therapy should be provided in patients with hypocalcemia before PTX to alleviate the postoperative complications of hypocalcemia. However, considering the small number of studies included in the meta-analysis, further studies with a larger sample size are still required to confirm this conclusion.

The authors discovered that the findings in some studies supported those in the majority of previous reports that younger patients with SHPT are at an increased risk of HBS [15,23,28,30]. They consider that a younger age is associated with a higher amount of sex hormone secretion, stronger osteoblast function, higher renal 1α-hydroxylase activity, and greater calcium utilization efficiency of bone tissue. This leads to the absorption of a large amount of serum calcium, causing relatively severe hypocalcemia after PTX [27,31]. Conversely, other researchers have reported the association between advanced age and HBS^19^. They considered that the higher prevalence of vitamin D deficiency and inadequate oral intake in elderly patients is associated with an increased risk of postoperative hypocalcemia [32]. However, the present meta-analysis revealed no association between age and postoperative hypocalcemia. To date, the consideration of age being a risk factor for hypocalcemia after PTX remains controversial, and more studies with larger sample sizes are necessary to verify this conclusion.

ALP activity is important for appropriate bone mineralization. In addition, it is also a well-recognized biomarker of renal osteodystrophy. Notably, ALP is the term given to a group of isoenzymes mainly found in the liver and bone. It is widely distributed in the skeletal system of the human body, with serum ALP level increasing upon activation of osteoblasts. Most patients with SHPT have metabolic bone diseases with active osteoblasts. In this case, the increased serum ALP level correlated with the severity of bone disease. On the contrary, higher ALP levels in patients with hypocalcemia before surgery suggest a more active bone remodeling state in patients with hypocalcemia after PTX [33,34]. Based on the results of this meta-analysis, it is concluded that a high preoperative ALP level was a risk factor for hypocalcemia after PTX. However, the meta-analysis exhibited strong heterogeneity. The very small OR value of multivariate logistic regression results in some studies is also a significant factor that led unsatisfactory results in the final meta-analysis. Despite the great heterogeneity of the meta-analysis, all the included studies consistently suggested that a high preoperative ALP level was a risk factor for postoperative hypocalcemia, thus confirming our conclusion. This preoperative variable allows physicians to accurately identify dialysis patients who are at a greater risk of hypocalcemia after PTX, and to aggressively monitor and treat patients who possess any of these factors in the postoperative period. An example of treatment is the supplementation of calcium and active vitamin D analogs.

Numerous studies have confirmed that preoperative iPTH concentrations and the magnitude of postoperative iPTH reduction in patients with HBS are higher than those in patients without HBS. This finding is consistent with the physiological functions of iPTH. Under stimulation by excessive iPTH, both bone formation and bone resorption exhibited an increase despite a marked negative balance [35–37]. Upon rapid decrease in iPTH level, the activity of osteoclasts *in vivo* decreases. This stops the osteolytic action, with a concurrent increase in osteoblast activity. A large amount of circulating calcium ions are then transferred to the bone to participate in osteogenesis. Thereafter, the serum calcium level declined after PTX, which was confirmed in a previous *in vitro* study [38–41]. Based on the results of this meta-analysis, it was concluded that a high preoperative iPTH level was a risk factor for postoperative hypocalcemia. Moreover, the reasons for high heterogeneity might be similar to that of ALP, including factors such as definition, surgical method, and continuous variables. However, all included studies consistently suggested that a high preoperative iPTH level was a risk factor for postoperative hypocalcemia, which also confirmed our conclusion. Clinicians should be aware of the important predictive role of preoperative iPTH level, better understand the postoperative course, and improve perioperative management to avoid the occurrence of hypocalcemia and minimize any adverse consequences.

Several limitations were noted in this meta-analysis. First, this was a retrospective observational study. Thus, the results were associated with possible selection bias and were limited by suboptimal data collection. Second, the potentially significant associations among variables could have been masked due to the relatively small sample size. Third, due to the heterogeneous definitions of significant postoperative hypocalcemia or HBS, results of this meta-analysis should be compared with other similar studies with caution. Fourth, most studies only focused on individual aspects such as the occurrence of early hypocalcemia immediately after the operation, whereas few studies addressed the problem from other perspectives, such as the length of postoperative hospital stay, hospital readmission, and total calcium requirement. Since these studies only concentrated on a particular area in the postoperative course, the data might not be reflective of the whole situation. Fifth, most of the 13 enrolled studies were from China, which might introduce geographical limitations in the results of this meta-analysis. Sixth, it is a pity that this review could not be registered online before the analysis.

## 5. Conclusions

Hypocalcemia after PTX is quite common, and the risk factors involved in its occurrence vary. In this study, a systematic search of major foreign medical databases was conducted, and 13 items were identified as possible risk factors for hypocalcemia in patients with SHPT after PTX. In conclusion, results of this meta-analysis show that preoperative serum Ca, ALP, and iPTH levels are significant risk factors for the occurrence of hypocalcemia after PTX. Full understanding of the conditions of individual patients and related risk factors should be possessed by medical staff. This allows early prediction and identification of high-risk groups and administration of scientific and targeted intervention measures which lead to improved standardization and effectiveness of disease diagnosis and treatment. Some examples of targeted intervention measures include early withdrawal of calcimimetics before surgery, monitoring of blood calcium level at intervals of 6 h after surgery, and early placement of intravenous access for high-dose calcium supplementation.

## Supplementary Material

Supplemental Material

Supplemental Material
