# Supplementary figures and images for: Risk factors for hypocalcemia in dialysis patients with refractory secondary hyperparathyroidism after parathyroidectomy: a meta-analysis
Source: Ren Fail. 2022 Mar 13;44(1):503–12. doi: 10.1080/0886022X.2022.2048856 (PMC8928856; doi:10.1080/0886022X.2022.2048856)

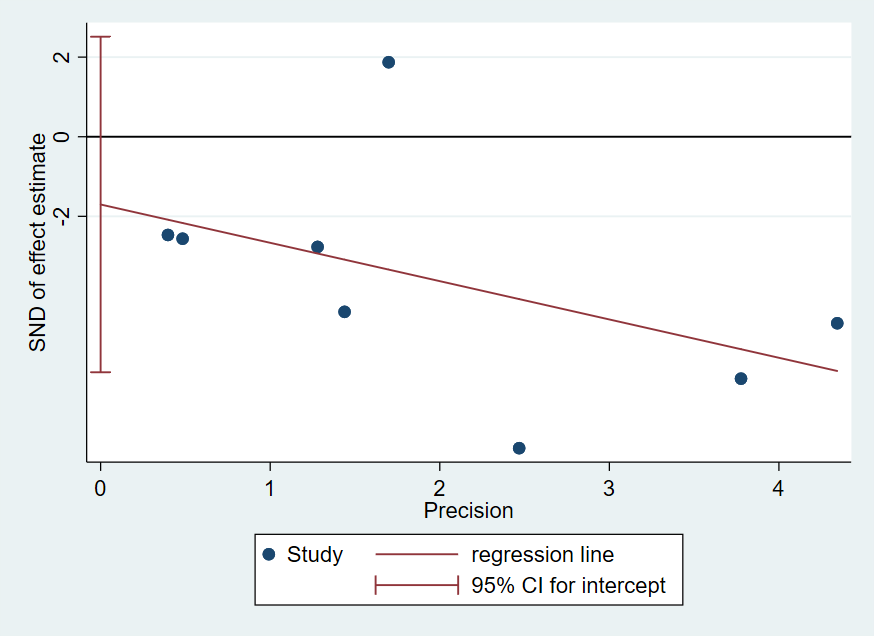

Supplement: Supplemental Material [file IRNF_A_2048856_SM1756.png]
