# Supplementary material for: Risk factors for hypocalcemia in dialysis patients with refractory secondary hyperparathyroidism after parathyroidectomy: a meta-analysis
Source: Ren Fail. 2022 Mar 13;44(1):503–12. doi: 10.1080/0886022X.2022.2048856 (PMC8928856; doi:10.1080/0886022X.2022.2048856)
Supplement: Supplemental Material [file IRNF_A_2048856_SM1749.pdf]

- The search terms used in PubMed were ((("Risk Factors"[Mesh]) OR (((((((((((Factor, Risk[Title/Abstract]) OR (Risk Factor[Title/Abstract])) OR (Health Correlates[Title/Abstract])) OR (Correlates, Health[Title/Abstract])) OR (Risk Scores[Title/Abstract])) OR (Risk Score[Title/Abstract])) OR (Score, Risk[Title/Abstract])) OR (Risk Factor Scores[Title/Abstract])) OR (Risk Factor Score[Title/Abstract])) OR (Score, Risk Factor[Title/Abstract])) OR (Population at Risk[Title/Abstract])) OR (Populations at Risk[Title/Abstract]))) OR ((relative[Title/Abstract] AND risk*[Title/Abstract]) OR (relative risk[Text Word]) OR risks[Text Word] OR cohort studies[MeSH:noexp] OR (cohort[Title/Abstract] AND stud*[Title/Abstract]))) AND (("Hyperparathyroidism, Secondary"[Mesh]) OR (((Secondary Hyperparathyroidism[Title/Abstract]) OR (Hyperparathyroidisms, Secondary[Title/Abstract])) OR (Secondary Hyperparathyroidisms[Title/Abstract]))).
- The search terms used in EMBASE were('secondary hyperparathyroidism'/exp OR 'secondary hyperparathyroidism':ab,ti OR 'hyperparathyroidisms, secondary':ab,ti OR 'secondary hyperparathyroidisms':ab,ti) AND ('Risk Factors'/exp OR 'Factor, Risk':ab,ti OR 'Risk Factor':ab,ti OR 'Health Correlates':ab,ti OR 'Correlates, Health':ab,ti OR 'Risk Scores':ab,ti OR 'Risk Score':ab,ti OR 'Score, Risk':ab,ti OR 'Risk Factor Scores':ab,ti OR 'Risk Factor Score':ab,ti OR 'Score, Risk Factor':ab,ti OR 'Population at Risk':ab,ti OR 'Populations at Risk':ab,ti).
